# Human Factor H-Related Protein 2 (CFHR2) Regulates Complement Activation

**DOI:** 10.1371/journal.pone.0078617

**Published:** 2013-11-18

**Authors:** Hannes U. Eberhardt, Denise Buhlmann, Peter Hortschansky, Qian Chen, Sascha Böhm, Markus J. Kemper, Reinhard Wallich, Andrea Hartmann, Teresia Hallström, Peter F. Zipfel, Christine Skerka

**Affiliations:** 1 Department of Infection Biology, Leibniz Institute for Natural Product Research and Infection Biology, Jena, Germany; 2 Department of Molecular and Applied Microbiology, Leibniz Institute for Natural Product Research and Infection Biology, Jena, Germany; 3 Pediatric Nephrology, University Medical Center Hamburg-Eppendorf, Hamburg, Germany; 4 Institute of Immunology, University of Heidelberg, Heidelberg, Germany; 5 Friedrich Schiller University, Jena, Germany; University of Leicester, United Kingdom

## Abstract

Mutations and deletions within the human *CFHR* gene cluster on chromosome 1 are associated with diseases, such as dense deposit disease, CFHR nephropathy or age-related macular degeneration. Resulting mutant CFHR proteins can affect complement regulation. Here we identify human CFHR2 as a novel alternative pathway complement regulator that inhibits the C3 alternative pathway convertase and terminal pathway assembly. CFHR2 is composed of four short consensus repeat domains (SCRs). Two CFHR2 molecules form a dimer through their N-terminal SCRs, and each of the two C-terminal ends can bind C3b. C3b bound CFHR2 still allows C3 convertase formation but the CFHR2 bound convertases do not cleave the substrate C3. Interestingly CFHR2 hardly competes off factor H from C3b. Thus CFHR2 likely acts in concert with factor H, as CFHR2 inhibits convertases while simultaneously allowing factor H assisted degradation by factor I.

## Introduction

The complement system serves as a central component of innate immunity, regulating hemostasis and cooperating with the adaptive immune response [1], [2]. The complement system is activated via three major pathways: the alternative (AP), classical (CP), and lectin (LP) pathway, which all lead to the generation of active C3 convertases. C3 convertases cleave C3 to form the opsonin C3b and the anaphylatoxin C3a. C3b attaches covalently to nearby surfaces and if not inactivated by regulators, forms C3 and C5 convertases. C5 convertases cleave C5 to C5b and the anaphylatoxin C5a, a critical step that initiates the terminal, lytic stage of complement. A fourth complement activation pathway - mediated through thrombin - directly activates C3 and C5 [1], [2].

Five factor H related proteins (CFHR1-CFHR5) circulate in human plasma, and three members of this group are described as complement regulators [3]. CFHR1 inhibits the C5 convertase and terminal complement complex (TCC) formation [4]. CFHR3 and CFHR5 act as cofactors for the serine protease factor I and both proteins promote the inactivation and degradation of C3b [5], [6]. CFHR4, in contrast is described as an activator of the complement system [7]. The role of CFHR2, the second member of the CFHR family in complement, is still unclear. CFHR2 is composed of four SCR domains and circulates in human plasma in both non-glycosylated and glycosylated forms [8]. The amino acid sequence of the two N-terminal SCRs of CFHR2 show almost sequence identity (98 and 100%) to the N-terminal SCRs of CFHR1. The two C-terminal SCRs of CFHR2 have 93 and 64% amino acid identity to SCRs 19–20 of factor H and to SCRs 4–5 of CFHR1. In factor H, these SCRs represent the combined C3b and surface recognition regions. Recently, homo- and hetero-dimerization of CFHR proteins was identified, mediated by a dimerization domain located in the N-terminal SCR domains of CFHR1, CFHR2 and CFHR5 [9].

Each single CFHR protein is encoded by a separate gene and all five *CFHR* genes are located on human chromosome 1q32 in the *CFHR* gene cluster [10], downstream of the *factor H* gene. Single nucleotide exchanges in this cluster, as well as chromosomal rearrangements and deletions are associated with several diseases, including atypical hemolytic uremic syndrome (aHUS), deficiency of CFHR proteins and autoantibody-positive HUS (DEAP-HUS), C3 glomerulopathy, IgA nephropathy, age-related macular degeneration (AMD), and systemic lupus erythematosus (SLE) [2], [3], [11]. Genomic rearrangements within the CFHR gene cluster that code for hybrid proteins were described for aHUS [12], [13], [14] and C3 glomerulopathy [15], [16]. Recently a CFHR2 polymorphism (rs3790414) was identified in an AMD cohort, which is correlated with the risk of developing neovascular AMD [17] and CFHR2 deficient patients were identified in an AMD patient cohort [18]. Additionally, Sethi et al. identified CFHR2 as component of glomerular dense deposits of a GN-C3 patient [19], which indicates that CFHR2 also plays a role in human diseases. Thus, there is a developing interest in the physiological role of CFHR2, as well as the other four CFHR proteins in complement activation.

CFHR2 binds to C3b [20], which suggests that CFHR2 has a functional role in the complement activation pathway [3], [8]. In the present study, we identify CFHR2 as a novel human alternative pathway complement regulator that inhibits the amplification loop of the AP at level of the C3 convertase and TCC assembly.

## Materials and Methods

### Proteins and serum

CFHR1 and CFHR2 were expressed as previously described [20]. CFHR2 deletion mutants were generated encoding the N-terminal SCRs (CFHR2/1–2) and C-terminal SCRs (CFHR2/3–4) using the following primers: CFHR2/1–2 Rev (5′-TCTAGAGCAGTGGACCTGCATTTGGG-3′) and CFHR2/3–4: Fwd (5′-GGTACCTCATTTCTGCAGAAAAATGTG-3′). The *CFHR2* gene products were amplified using liver cDNA (Life Technologies), cloned into the pPICZαB vector (Life Technologies), and expressed in *P. pastoris* (strain X33) according to standard protocols. Expressed His-tagged proteins were purified by Ni^2+^-chelate affinity chromatography as previously described [4] followed by gel chromatography. C3dg was cloned by using the following primer pair: Fwd CACCGAAGGAGTACAGAAAGAGGA and Rev TTAACGAGAAGGCAGCTGCAGA and expressed by the *E.coli* pET200/D-Topo expression system (Life Technologies). Purification was performed by applying Ni-NTA Superflow Cartridge (Qiagen) affinity chromatography according to the supplier recommendations. Purified C3dg was concentrated using Amicon Ultra-15 filters (Merck Millipore) and dialyzed against DPBS (Lonza). Absolute molecular masses were determined by static light scattering experiments using a miniDawn TREOS monitor in combination with an Optilab T-rEX differential refractometer (Wyatt). Proteins were applied on HiLoad 16/60 Superdex 200 pg (CFHR2), Superdex 200 10/300 GL (CFHR2/1-2) and HiLoad 16/60 Superdex 75 pg (CFHR2/3-4) columns (GE Healthcare). Identified peaks were processed by ASTRA 6 software (Wyatt). Absolute molecular masses were calculated from both the refractive index and light scattering responses. Proteins were separated by SDS- PAGE and visualized either by silver staining or immunoblotting using standard methods.

Factor H, I, D, B, vitronectin (Vn), C3, C3b, C3d, C3d and C3a antiserum were purchased from Complement Technology (Tyler, USA) and CRIg from Eton Bioscience (San Diego, USA). Normal human serum (NHS) was obtained from healthy donors with informed consent.

### Binding assays

#### Surface plasmon resonance

Real-time binding analysis was performed using a Biacore T200 system (GE Healthcare) at 25°C. Data were processed with Scrubber 2.0c (BioLogic Software). C3b (1 µM) was biotinylated with Sulfo-NHS-LC-Biotin (Pierce) by using a 1∶1 molar ratio in 10 mM HEPES pH 7.4, 150 mM NaCl, 50 µM EDTA, 0.005% (v/v) surfactant P20 (HBS-EP) at 4°C for 2 hours. N-biotinylated C3b was injected on flow cell 2 of a streptavidin (Sigma)-coated CM3 sensor chip at a flow rate of 10 µl/min (520 RU). Factor H or CFHR2 (each 6.25–3200 nM in HBS-EP) association and dissociation times were set to 120 seconds (CFH) or 60 seconds (CFHR2) at a flow rate of 30 µl/min. Each injection was performed at least 3 times. Regeneration was achieved with 20 mM sodium acetate, 1 M NaCl, pH 4.0 for 30 seconds. Refractive index errors due to bulk solvent effects were corrected with responses from non-coated flow cell 1 as well as subtracting blank injections. K_D_ values were calculated from the kinetic rate constants for CFH- C3b or CFHR2-C3b complex formation and dissociation derived from a 1∶1 interaction model including a mass transport term.

#### ELISA

C3 activation products C3b, C3c, C3dg and C3d (each 142 nM) (Comptech except C3dg) were coated onto MaxiSorp microtiter plates (Nunc) and incubated with CFHR2 or factor H (each 0.07 µM). Binding was detected with CFHR1 and factor H antisera. For competition assays C3b or C3dg (5 µg/ml) was immobilized and incubated with constant amounts of factor H (10 µg/ml≙0.07 µM) and increasing amounts of CFHR2 (2.5–40 µg/ml≙0.035–0.56 µM) or with constant amounts of CFHR2 (5 µg/ml≙0.07 µM) and increasing amounts of factor H (5–50 µg/ml≙0.033–0.33 µM). Bound factor H was detected using factor H/1-4 antiserum, that does not crossreact with CFHR2, and monoclonal antibody A 72 which does not detect factor H (generated with standard methods).

### Complement activation assays

Complement activation assays were performed as previously described [21]. NHS was diluted in Mg-EGTA buffer (20 mM HEPES, 144 mM NaCl, 7 mM MgCl_2_, and 10 mM EGTA, pH 7.4) or GVB++ buffer (Complement Technologies). NHS (20% for AP, 1% for CP) was preincubated for 15 min at 37°C with CFHR2, CFHR2/1–2, CFHR2/3–4, factor H, C4BP, or BSA (all 5–25 µg/ml) and added to microtiter wells pre-coated with either LPS (10 µg/ml) or IgM (2 µg/ml) for 1 h at 37°C. Complement activation was measured using anti-human C3b (Fitzgerald) or C5b-9 (Dako) monoclonal Abs. For comparison CFHR2, factor H and CRIg (each 0.001–1 µM) were added to NHS (20% in HEPES-EGTA buffer) incubated with LPS coated wells as described and complement activation was followed by C3b deposition and detection of C3a or Ba in the supernatant by ELISA Kits (Quidel). In addition C3b deposition was followed by similar assays and in this case CFHR2 and factor H were used together (each 0.01–2.5 µg/ml). The AP C3 convertase, C3bBb, was assembled according to the method as described [22] in the presence of CFHR2 (1–50 µg/ml≙0.014–0.7 µM), factor H or BSA (each 50 µg/ml), followed by the addition of factor D (4 µg/ml). C3a generation was quantified by immunoblotting using polyclonal C3a antiserum.

The TCC formation was followed on sheep red blood cells (SRBC) in AP buffer (20 mM HEPES, 144 mM NaCl, 7 mM MgCl_2_, 10 mM EGTA, pH 7.4). SRBC (5×10^6^; Bio Trend) were loaded with C5b6 (3 µg/ml) for 10 min at RT. In the meanwhile CFHR2, CFHR2/1-2, CFHR2/3-4 (1–20 µg/ml), factor H (40 µg/ml) and vitronectin (Vn) (40 µg/ml) were preincubated with C7 (2 µg/ml), C8 (2 µg/ml) and C9 (2 µg/ml) for 5 min at 37°C. Preincubated protein mixtures were incubated for 30 min at 37°C with the C5b6 loaded SRBC, and the SRBC lysis quantified by measuring the absorbance of the supernatant (5 min, 800× g) at 414 nm.

### C3 decay acceleration and cofactor assays

To determine decay accelerating activity purified C3b (2.5 µg/ml) was immobilized on a microtiter plate and the C3bBb(Mg^2+^) complex generated by addition of factor B (0.5 µg/ml) and factor D (25 ng/ml) in Mg-PBS buffer (½ PBS, 10 mM MgCl_2_, 4% (v/v) BSA). CFHR2 (1–100 µg/ml) or factor H (1–10 µg/ml) was incubated with the preformed convertase for 1 h at RT, and convertase stability was followed by measuring C3b bound factor B using a factor B Ab (Calbiochem).

Cofactor activity of CFHR2 for the degradation of C3b in fluid phase was performed by incubation of C3b (8 µg/ml), factor I (4 µg/ml), different amounts of CFHR2 (1–100 µg/ml≙0.014–1.4 µM), and factor H (0.8 µg/ml≙0.05 µM) for 30 min at 37°C. Reactions were stopped by adding SDS sample buffer, and samples were immuno-blotted with polyclonal C3 antiserum (Complement Technologies).

### Statistical analysis

Significant differences between two groups were analyzed using the unpaired Student's t-test. Values of *p≤0.05, **p≤0.01, ***p≤0.001 were considered statistically significant.

## Results and Discussion

### Recombinant CFHR2 forms exclusively homodimers

**Figure 1:**
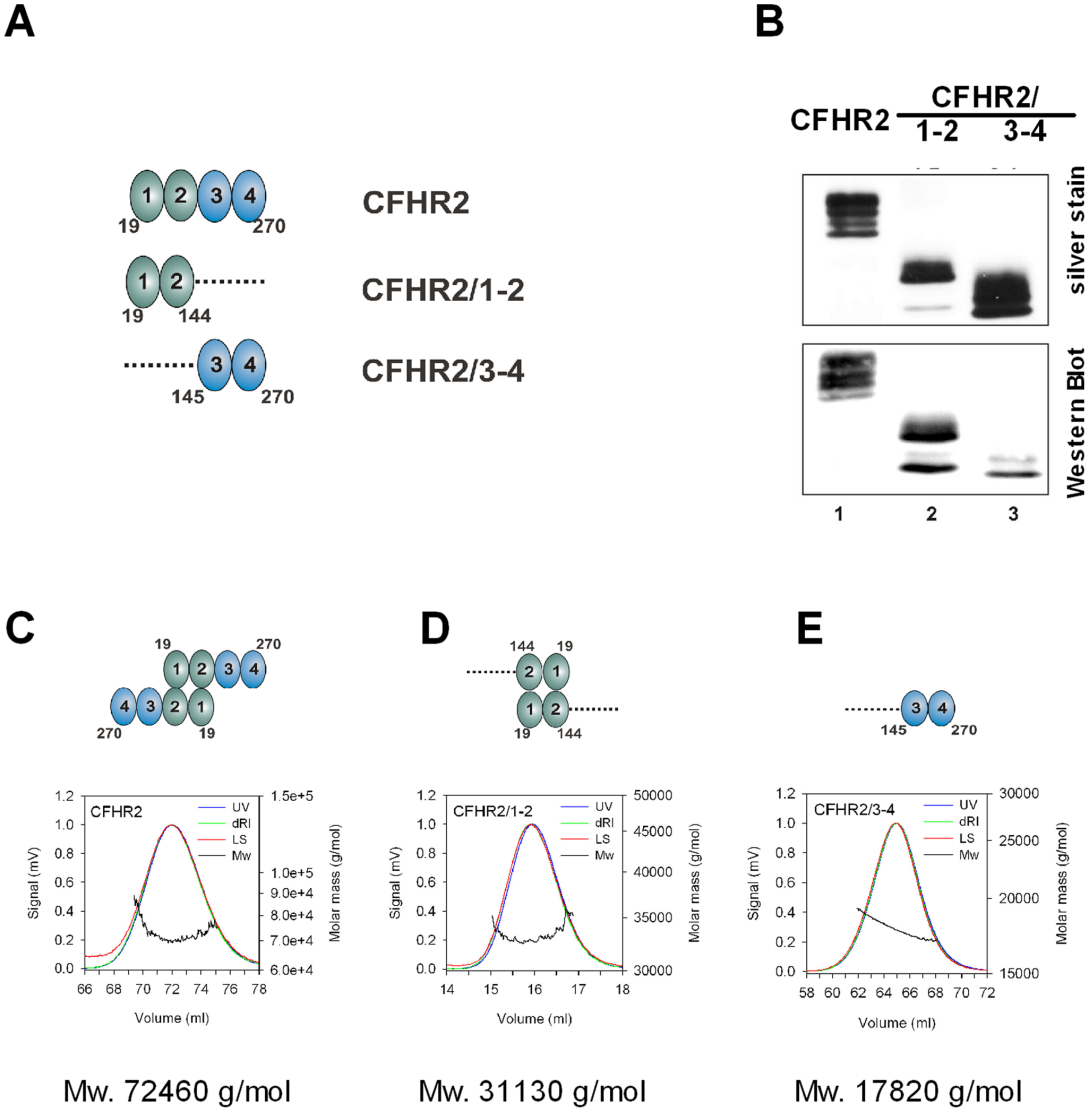
CFHR2, CFHR2/1-2 and CFHR2/3-4 protein expression and purification. (**A**) Domain composition of CFHR2 and CFHR2 fragments CFHR2/1-2 and CFHR2/3-4. Numbers indicate amino acids. (**B**) Recombinant purified full length CFHR2 and two CFHR2 fragments (CFHR2/1-2 and CFHR2/3-4) were separated by SDS-PAGE and detected by silver staining and Western blot using CFHR2 specific antiserum. CFHR2 appeared with mobility of ∼27 to 33 kDa, and both CFHR2 mutants of ∼16 to 18 kDa. (**C**) Determination of the solution oligomeric state of CFHR2 proteins. Recombinant CFHR2 proteins were purified by Ni^2+^-chelat chromatography, deglycosylated and purified by gel chromatography (Superdex 200 or Superdex 75). Analysis of the solution oligomeric state of CFHR2, (**D**) CFHR2/1-2 and (**E**) CFHR2/3-4 by static light scattering in combination with size exclusion chromatography. The light scattering signals (LS) are shown as the mass distribution (Mw) in each of the peaks in the elution profiles monitored by the absorbance at 280 nm (UV) and changes of the refractive index (dRI). The molar mass of each protein is indicated as well as the status of dimerization.

To identify the role of CFHR2 in complement activation we expressed CFHR2 and CFHR2 fragments (Fig. 1A) recombinantly in *Pichia pastoris*. CFHR2 proteins were isolated and purified from the cell supernatants. Subsequent to SDS-PAGE separation, and followed by silver staining or immune-blotting the purified full length CFHR2 protein appeared as several bands with mobilities ranging from 28 to 30 kDa (Fig. 1B). These bands represent distinct glycosylated forms of CFHR2, as recombinant CFHR2 when treated with endoglycosidase, resulting in one single band with a mobility of 28 kDa [8]. Purification by gel chromatography showed a recombinant, non-reduced CFHR2 which eluted at a molecular mass of 72 kDa and which existed exclusively as a homodimer (72 kDa) (Fig. 1C). In addition the N terminal CFHR2/1-2 fragment when also investigated by gel chromatography eluted at a molecular mass of 33 kDa (Fig. 1D), which was double that of the expected mass of 17 kDa. In contrast, the CFHR2/3-4 fragment eluted as a monomeric 17 kDa fragment (Fig. 1E). Thus, recombinant CFHR2 forms homodimers and the N-terminal two SCRs mediate this dimerization. These results are in agreement with the recently identified dimerization domain in SCRs 1–2 of CFHR1, CFHR2 and CFHR5 [9], [16].

### CFHR2 binds to C3b

**Figure 2:**
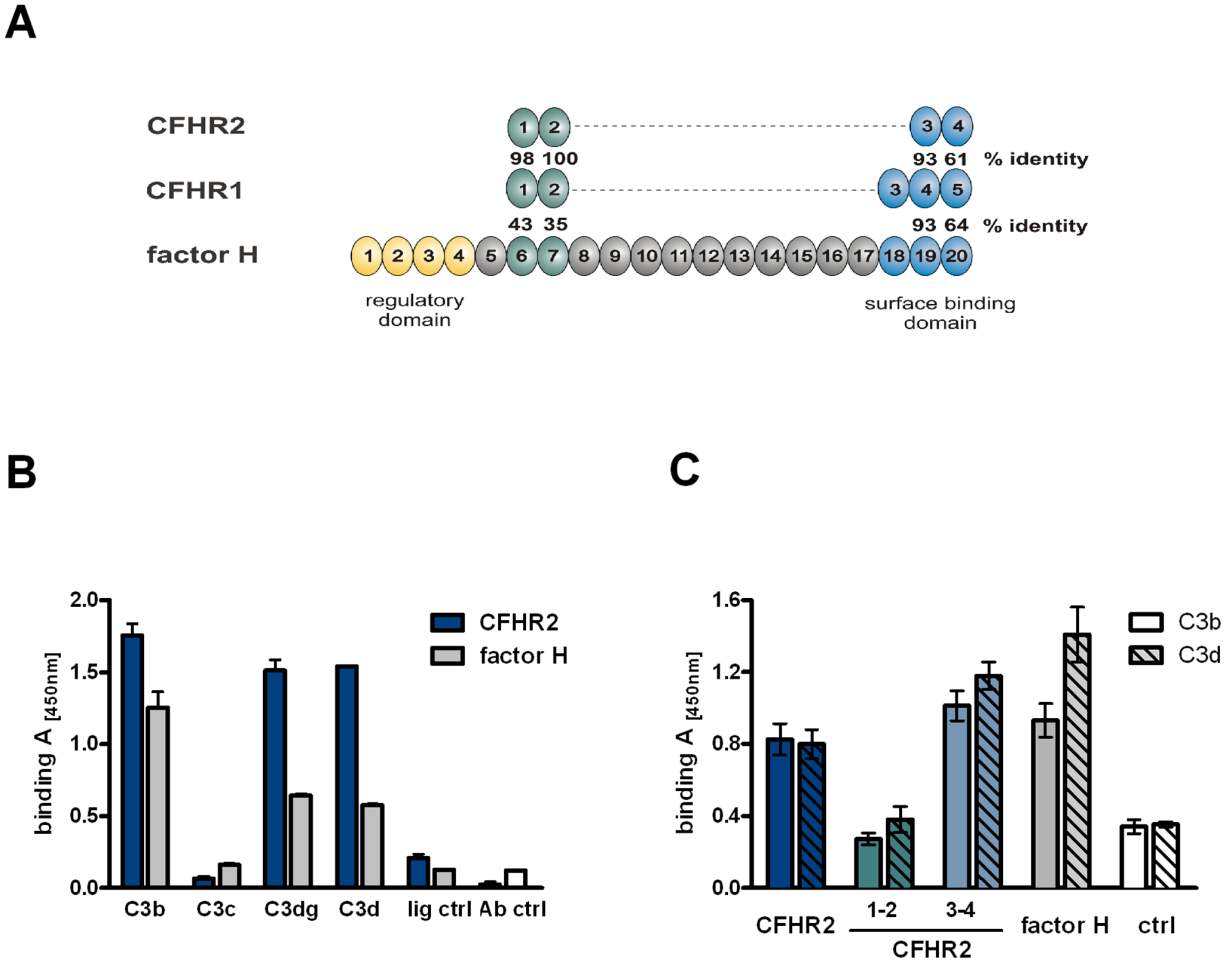
CFHR2 binds via the C-terminus to C3b and C3d. (**A**) CFHR2 SCR domains are aligned against the highly related SCR domains of CFHR1 and factor H. The number above each SCR indicates the identity to the corresponding domain in CFHR1 respectively factor H. The regulatory region (yellow), SCRs 6–7 (green), and the surface recognition site (blue) of factor H are marked. (**B**) CFHR2 binds to C3b, C3dg and C3d, but not to C3c. In parallel factor H binding was determined. Equimolar amounts of CFHR2 and factor H were used. Data represent mean values ± SD of four independent ELISA experiments. Background binding of the antibodies to ligand C3b alone (lig ctrl) or without ligand C3b (Ab ctrl) is shown. (**C**) CFHR2 binds via the two C-terminal domains to C3b and C3d. The proteins were used in equimolar amounts. Data represent mean values ± SD of three independent experiments. Background binding of the antibodies to ligand C3b or C3d alone (ctrl) is shown.

**Figure 3:**
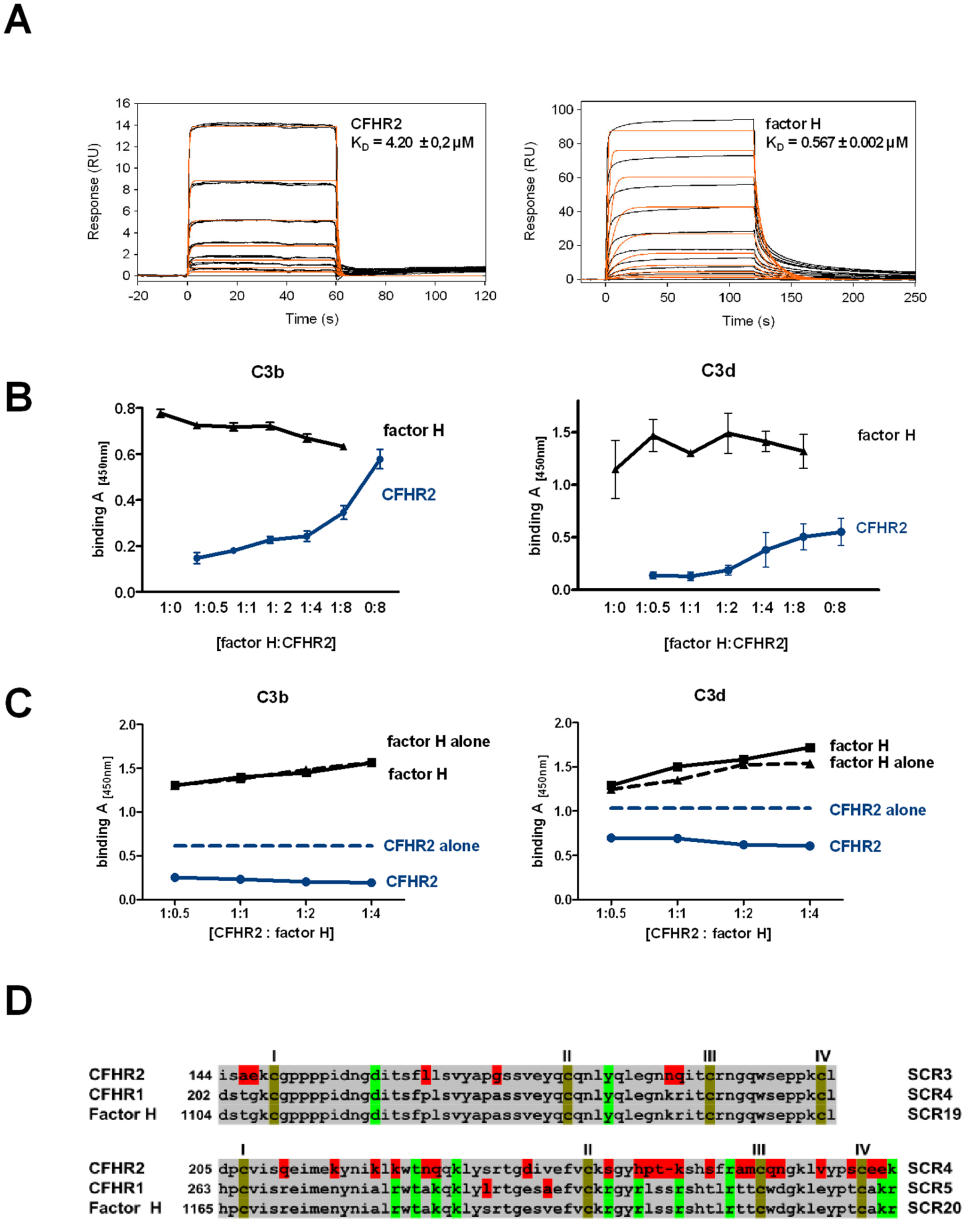
CFHR2 binds to C3b and C3d and does not compete off factor H. (**A**) Real-time *in vitro* SPR binding analysis of CFHR2 (left panel) and factor H (right panel) to sensor immobilized C3b. CFHR2 and factor H binding sensograms (black lines) are shown overlaid with the best fit derived from a 1∶1 interaction model including a mass transport term (red lines). As CFHR2 forms exclusively dimers, one CFHR2 dimer was regarded as one molecule. (**B**) CFHR2 slightly competes off factor H from binding to C3b (left panel) and does not effect factor H binding to C3d (right panel). Mean values ± SD of three independent experiments and the molar ratios of factor H (1 = 66 nM) to CFHR2 are shown. (**D**) factor H binds preferentially to C3b and to C3d in the presence of constant amounts of CFHR2 (1 = 66 nM). Constant amounts of CFHR2 and increasing concentrations of factor H were incubated with immobilized C3b or C3d. Binding rates of CFHR2 and factor H alone to C3b or C3d are represented by dashed lines. A representative experiment is shown. (**E**) C-terminal amino acid sequence alignment of CFHR2 SCR3 and SCR4 with factor H SCR19 and SCR20. Conserved cysteins (I–IV, brown), identical (grey) and non-identical amino acids (red) are marked. Charged amino acids that are relevant for C3b and heparin binding are indicated (green).

The C-terminal two SCRs, ie. SCR3 and SCR4 of CFHR2 show high, as well as moderate sequence identity to the C-terminal C3d binding SCRs, domains SCRs 19 (93%) and 20 (63%) of factor H (Fig. 2A). This identity suggests that CFHR2 binds via the two C- terminal SCRs to C3b/C3d and therefore CFHR2 binding to C3 cleavage products was investigated by ELISA. Recombinant CFHR2 bound to C3b, C3d and to C3dg, but not to C3c (Fig. 2B). The binding site in CFHR2 was localized by using the N- and C-terminal fragments in the binding assays. The N-terminal fragment CFHR2/1-2 did neither bind to C3b nor to C3d and the C-terminal fragment, CFHR2/3-4 bound to both C3b and C3d (Fig. 2C). Thus a CFHR2 dimer harbors two binding sites for C3b or C3d. To further characterize this interaction and to define binding affinities, C3b was coupled to a sensor chip, and binding of CFHR2 was determined by surface plasmon resonance (SPR). CFHR2 bound to immobilized C3b with a K_D_ of 4.2 µM, and in the same setting factor H bound to C3b with a K_D_ = 0.57 µM (Fig. 3A). Hence CFHR2 bound with about 10 fold lower affinity as factor H to immobilized C3b.

To investigate whether CFHR2 and factor H bind to the same or to distinct regions in C3b or in C3d, the effect of CFHR2, used at increasing levels, on factor H binding to immobilized C3b and C3d was analyzed by ELISA. CFHR2 and factor H were detected with specific antibodies, CFHR2 with the specific monoclonal ab A72, and factor H with SCR1-4 antiserum, which recognizes factor H, but not CFHR2. In the presence of factor H, CFHR2 bound dose dependently to C3b, but CFHR2 did not significantly influence factor H binding to C3b (∼15% at 1 µM) (Fig. 3B). These results suggest that CFHR2 and factor H bind either to distinct sites in C3b or that CFHR2, due to the lower affinity for C3b as compared to factor H, does not compete off factor H from C3b. These results are supported by recently published experiments, which at physiological concentrations also show low competition between CFHR2 SCRs 3–4 and factor H [9]. Lack of factor H competition by CFHR2 contrasts to the roles of CFHR1 and CFHR3, which compete off factor H from C3b [4]. CFHR2 also did not replace factor H from C3d (Fig. 3B). Factor H contacts C3d with SCR19 and SCR20 and the two residues D1119 and Q1139 in SCR19 as well as the five residues in SCR 20 (i.e. K1186, R1203, R1206, R1210 and K1230) seem relevant for C3d binding (Fig. 3D) [23], [24]. The two relevant residues in SCR19 of factor H are conserved in the corresponding SCR3 of CFHR2. However, SCR4 of CFHR2 lacks the five relevant amino acid residues in SCR20 of factor H. This significant amino acid difference in SCR4 of CFHR2 may explain the distinct binding activities of factor H and CFHR2. To further characterize CFHR2 and factor H binding to C3b and C3d, CFHR2 used at constant levels was added to factor H, which was used in increasing concentrations. The mixture was bound to immobilized C3b or to C3d and after washing, bound CFHR2 and factor H were determined. Factor H binding to C3b and C3d increased in a dose dependent manner and factor H binding to C3b and C3d was constant in the presence or absence of CFHR2 (Fig. 3C). These results are in agreement with the higher affinity of factor H for C3b as compared to CFHR2. The CFHR2 signals remained almost constant in the presence of factor H. However, in the absence of factor H more CFHR2 bound to C3b and to C3d (Fig. 3C). Thus, indicating that factor H interferes CFHR2 binding to both C3b and to C3d. In summary, CFHR2 does not compete off factor H neither from C3b nor from C3d, but factor H may affect CFHR2 binding to C3b and to C3d.

### CFHR2 inhibits the alternative complement pathway

**Figure 4:**
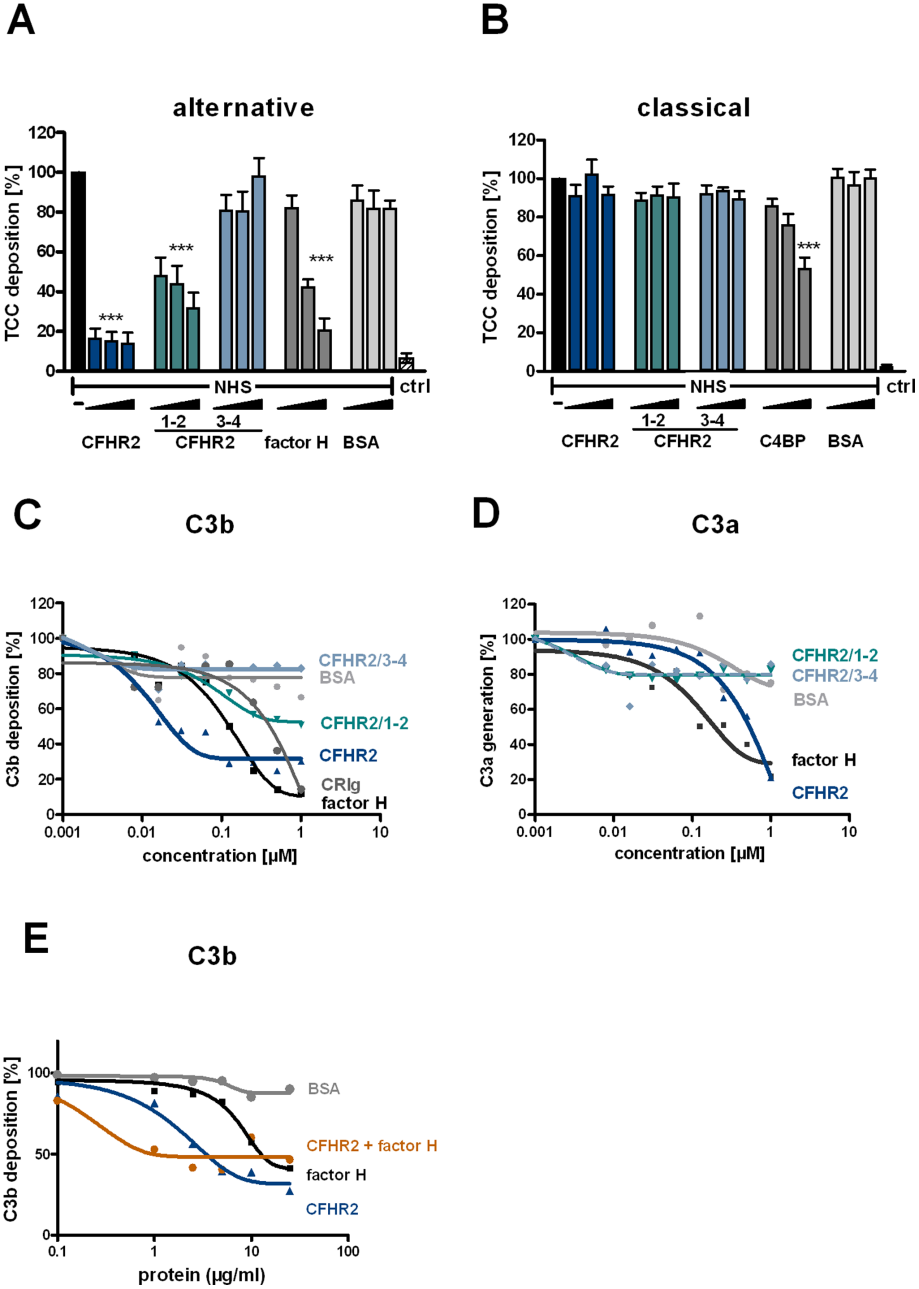
CFHR2 regulates AP complement activation. (**A**) CFHR2 inhibits AP activation in NHS. (**B**) CFHR2 had no effect on CP activation in NHS. TCC deposition in untreated NHS was set as 100%. C4bp is a classical pathway inhibitor. (**C**) CFHR2 and CFHR2/1–2 but not CFHR2/3–4 inhibited C3b deposition. (**D**) C3a generation in NHS activated via AP (ELISA). C3b depositon in untreated NHS was set as 100%. (**E**) CFHR2 together with factor H increased inhibition of C3b deposition in NHS as compared to CFHR2 or factor H alone. Data in (**A–E**) represent the mean values ± SD of three independent experiments (^*^p≤0.05, ^***^p≤0.001). Control (ctrl) indicates binding of the detection Ab to the plate.

Knowing that CFHR2 binds to C3b, we asked whether CFHR2 acts as a complement regulator and therefore we assayed if purified, recombinant CFHR2 affects either the AP or CP. CFHR2 was added to normal human serum (NHS), complement was activated via the AP by LPS and terminal complement complex (TCC) deposition was followed with a C9 neoepitope recognizing antibody. CFHR2 inhibited TCC surface deposition (Fig. 4A). The dimeric N-terminal fragment CFHR2/1-2 also inhibited AP complement activation, but the C-terminal monomeric fragment CFHR2/3-4 lacked this activity (Fig. 4A). In this set up CFHR2 did not influence TCC surface deposition when complement was activated via the CP (Fig. 4B). Thus CFHR2 inhibits the AP and the N-terminal SCRs are involved in this regulatory effect.

### CFHR2 inhibits the C3 convertase

To define at which step CFHR2 inhibits the complement cascade, we assayed CFHR2 effects on the earlier cascade steps. Therefore CFHR2 was added to NHS, complement was activated via the AP, and C3 convertase activity was evaluated by following C3b deposition. CFHR2 used at 0.1 µM inhibited C3b deposition by about 75% (Fig. 4C). The N-terminal deletion mutant CFHR2/1-2, but not the CFHR2/3-4 fragment reduced C3b deposition. However, in this case CFHR2/1-2 had a much lower inhibitory effect. Intact, full length CFHR2, and also the regulators factor H and CRIg [25] showed comparable inhibitory activities (Fig. 4C).

As CFHR2 bound to C3b and reduced C3b deposition an inhibition at the level of the C3 convertase was concluded. To further evaluate this inhibitory role, the effect of CFHR2 on the C3 convertase mediated C3 cleavage was evaluated by following C3a generation. CFHR2 was added to NHS, complement was activated as before via the AP and C3a levels were determined in the supernatant by ELISA. CFHR2 inhibited C3a generation and this effect was dose dependent and was comparable to the effect of factor H. In this case the CFHR2/1-2 fragment lacked the inhibitory activity (Fig. 4D). Thus the N-terminal fragment of CFHR2 does not inhibit C3 cleavage.

As both CFHR2 and factor H bind to C3b (Figure 3) and inhibit C3 convertase activities we assayed how the combination of the two proteins modulates complement activation. To this end CFHR2 and factor H, each used at concentration ranging from 0.01 to 2.5 µg/ml, were combined, then added together to NHS and this mixture to LPS coated microtiter plates. After incubation and washing, C3b deposition was followed by ELISA. At low concentrations (each 1 µg/ml) the combination of CFHR2 and factor H inhibited complement activation and reduced C3b deposition about 50%. CFHR2 used alone at 1 µg/ml reduced C3b deposition by about 20% and factor H at 1 µg/ml by about 10% (Figure 4E). These results indicate that CFHR2 and factor H can inhibit complement activation in concert.

### CFHR2 inhibits the *in vitro* assembled C3 convertase

**Figure 5:**
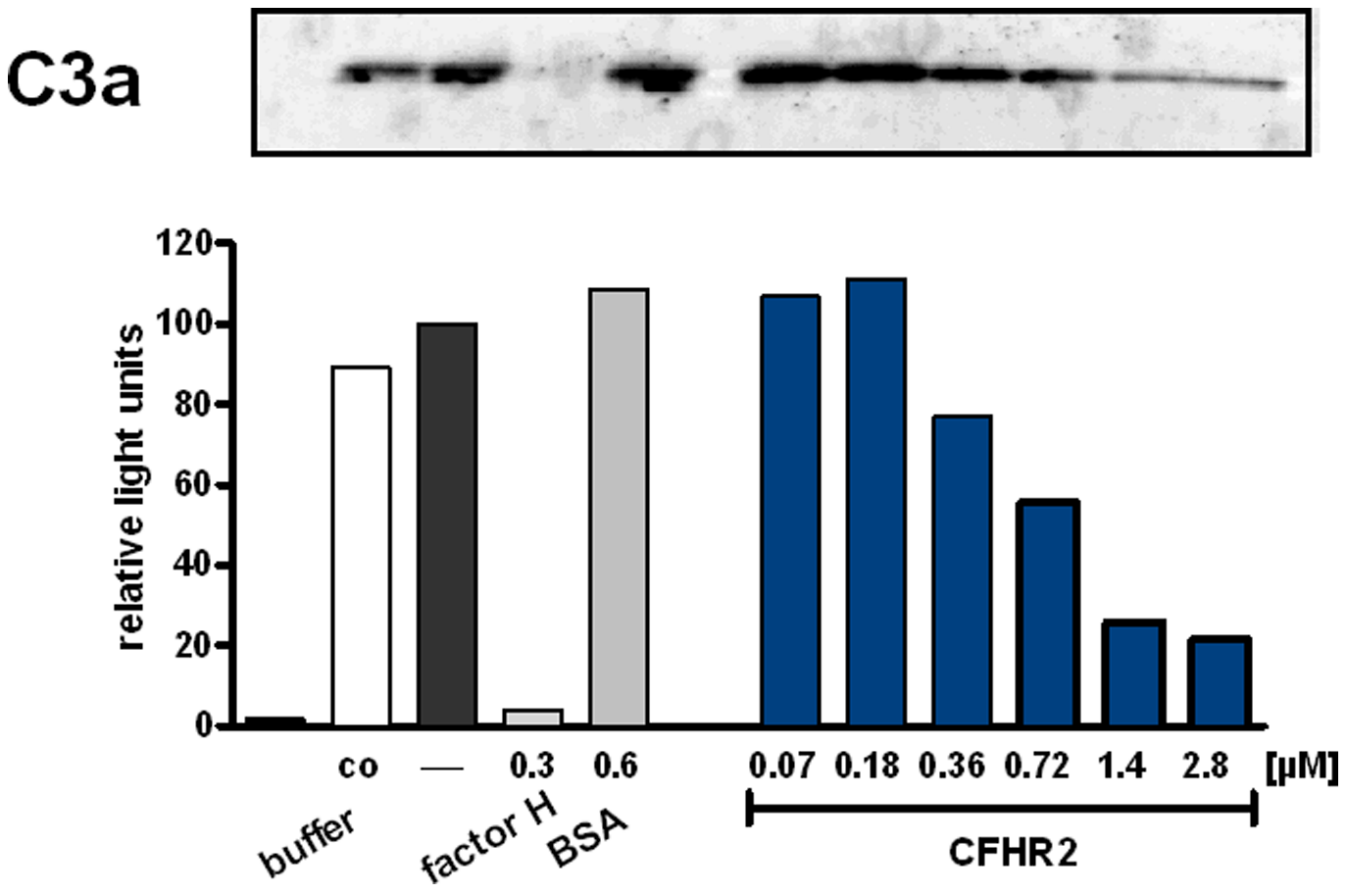
CFHR2 inhibits the AP C3 convertase. CFHR2 inhibited *in vitro* assembled AP C3 convertase as measured by C3a generation. The corresponding densitometric analysis of the C3a bands of the Western blot is shown. C3a generation by C3 convertase alone was normalized to 100%. A representative analysis of three experiments is shown.

To define in more detail how CFHR2 controls the C3 convertase, the inhibitory effect of CFHR2 was followed on an AP C3 convertase that was assembled *in vitro* with purified proteins. In this case C3a generation was followed. CFHR2 inhibited C3a generation and the densitometric analysis of the C3a Western Blot showed for CFHR2 a dose dependent effect for C3a release (Fig. 5).

**Figure 6:**
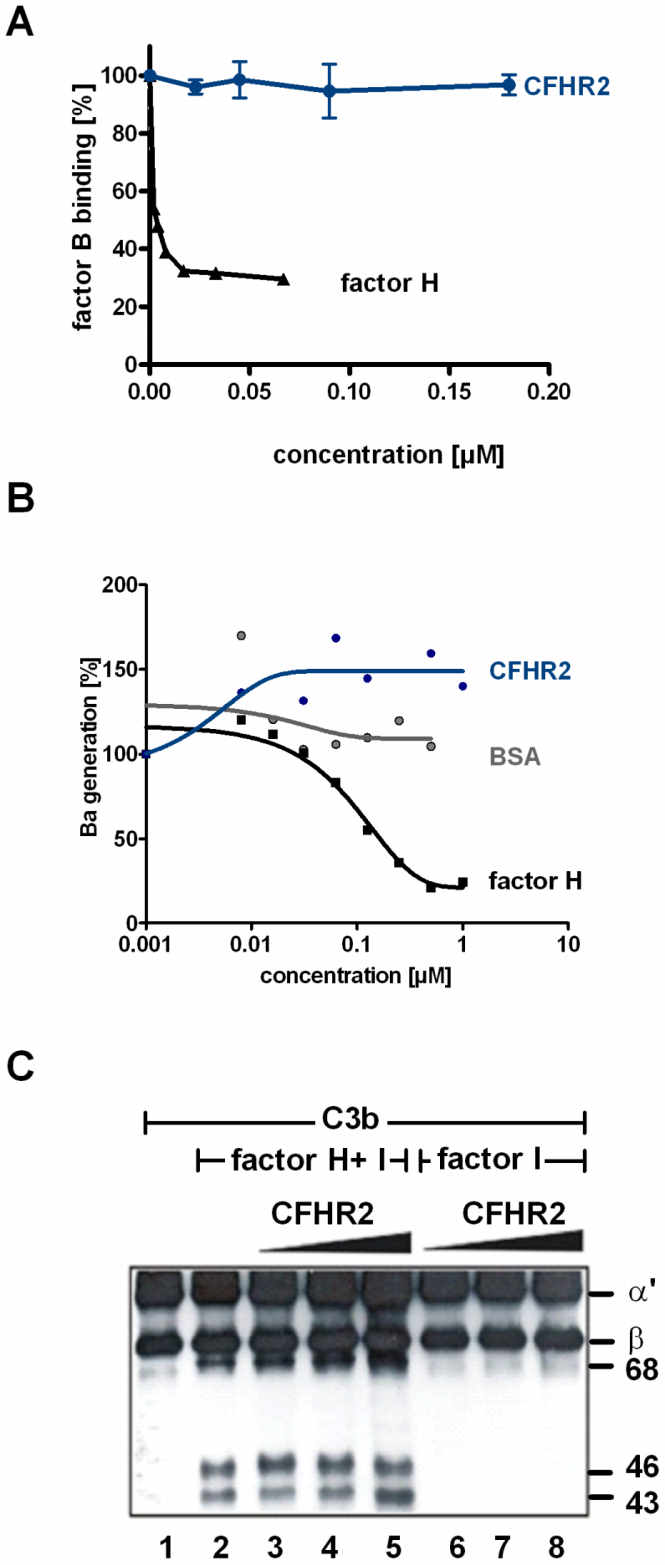
CFHR2 exerts neither decay acceleration nor cofactor activity. (**A**) CFHR2 can not dissociate factor B from the *in vitro* assembled convertase C3bBb, but factor H efficiently decays factor Bb fragment from C3b. (**B**) CFHR2 does not influence cleavage of C3b bound factor B by factor D. CFHR2 preincubated NHS, which was activated by surface immobilized IgM via the AP and Ba cleavage was followed by ELISA. A representative experiment is shown. (**C**) CFHR2 alone lacks cofactor activity for factor I to degrade C3b (lanes 6–8). CFHR2 does not interfere with factor H cofactor activity (lanes 2–5). Increasing amounts of CFHR2 (1–100 µg/ml) were incubated with C3b and factor I in the presence or absence of factor H. Reaction mixtures were separated by SDS PAGE and immunoblotted using a C3 polyclonal antibody.

To further characterize how CFHR2 influences C3 convertase activity, we investigated whether CFHR2, similar to factor H, exerts decay accelerating activity. To this end we analyzed if CFHR2 dissociates Bb from the preformed C3 convertase. CFHR2 did not, but factor H did dissociate factor B/Bb from the convertases. This differences to factor H reveals that CFHR2 inhibits the C3 convertase by a different mechanism than by decay accelerating activity (Fig. 6A). Therefore we next assayed whether CFHR2 inhibits the C3 convertase by influencing cleavage of factor B bound to C3b by factor D. Again CFHR2 was added to NHS, AP was activated and generation of Ba was determined. In this assay CFHR2 did not, but again factor H reduced Ba generation (Fig. 6B).

### CFHR2 lacks cofactor activity for factor I

Several complement regulators act as cofactors and allow degradation, processing and inactivation of newly formed C3b by the serine protease factor I. To determine whether CFHR2 mediates such a cofactor activity for factor I, CFHR2 was added to C3b and factor I, and after incubation degradation of C3b was assayed by immuneblotting. In the presence of CFHR2, C3b remained intact and no degradation occurred (Fig. 6C). As expected factor H displayed cofactor activity and C3b degradation products with mobilities of α′68, α′46 and α′43 kDa were detected. When CFHR2 was added in combination with factor H and factor I to C3b, cleavage of C3b by factor I was neither reduced nor enhanced. Thus CFHR2 lacks cofactor activity and did not influence the cofactor factor H for factor I in C3b degradation. These results also confirm that CFHR2 does not compete off factor H bound to C3b.

### CFHR2 inhibits the assembly of the terminal complement complex (TCC)

**Figure 7:**
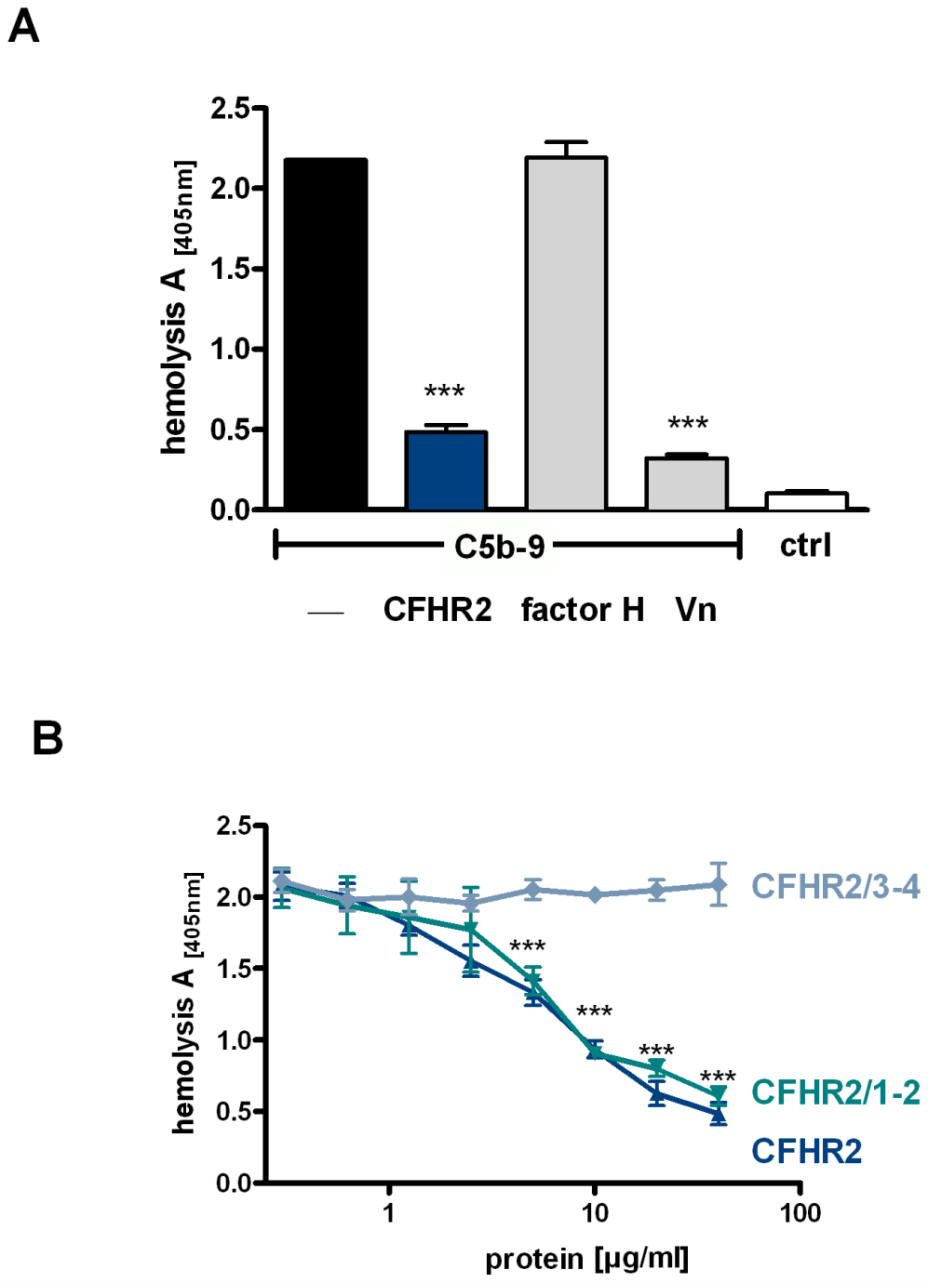
CFHR2 inhibits terminal pathway activation. (**A**) CFHR2 (blue column), factor H (gray column) and vitronectin (Vn, gray column) were preincubated with purified TCC components C7, C8,C9 and added to C5b-6 loaded SRBC. TCC mediated erythrocyte lysis was monitored by measuring the absorbance of the supernatant at 414 nm. Reduced absorbance correlates with inhibition of the TCC formation on SRBC. (**B**) CFHR2 inhibited TCC formation via the N-terminal SCRs 1–2 in a dose dependent manner. CFHR2 proteins were preincubated with TCC components and lysis of SRBC was measured. Data in (**A**) and (**B**) represent mean values ± SD of three independent experiments (^***^p≤0.001).

To further characterize the role of CFHR2 in complement activation, the effect of CFHR2 on the terminal pathway activity was assayed using purified proteins, i.e. C5b6, C7, C8. CFHR2 was first added to C5b6 and C7; then this mixture was combined with sheep red blood cells (SRBC) and C8 and C9 were added. Following incubation TCC generation was evaluated by recording erythrocyte lysis. CFHR2 added at 20 µg/ml (0.29 µM) inhibited erythrocyte lysis and this effect was comparable to that of the soluble human TCC inhibitor vitronectin (VN) (Fig. 7A). As expected factor H (40 µg/ml) did not block TCC formation.

To localize the domain of CFHR2 that mediates this TCC regulatory activity, the two CFHR2 deletion fragments were tested in the same assay. CFHR2/1-2, but not the C-terminal CFHR2/3-4 inhibited TCC formation (Fig. 7B). This inhibitory effect was dose dependent. The N-terminal fragment and the full length CFHR2 protein showed comparable activities (Fig. 7B). Thus CFHR2 regulates TCC assembly and the dimeric N-terminal SCR domains 1–2 mediate this inhibitory effect. Similar to CFHR2 also CFHR1, which shares a high amino acid identity with the two N-terminal domains, i.e. SCR1 and SCR2 of CFHR2 (97% and 100%, respectively) acts as a TCC inhibitor [4].

## Conclusions

The human plasma protein CFHR2 forms homodimers, binds to C3b and to C3d and inhibits the AP C3 convertase of complement. As CFHR2, lacks both cofactor and decay accelerating activities this homodimer apparently inhibits the C3 convertase by an other mechanism. The CFHR2 dimer, which is linked by the N terminal two SCRs exposes two separate C terminal C3b or C3d binding regions. Thereby one CFHR2 dimer can bind to two separate C3b proteins or even two C3 convertases and this linkage may explain why CFHR2 inhibits cleavage of the substrate C3 by the C3 convertase. So far such an inhibition of C3 substrate cleavage of the C3 convertase is only described for CRIg. However, CRIg by binding to the β-chain of C3b inhibits C3 substrate binding and cleavage [25]. Dimerization of the intact, full length CFHR2 is necessary for this C3 convertase inhibitory effect as both the N-terminal two SCRs i.e. CFHR2/1-2 and also the two C-terminals SCRs, CFHR2/3-4 lacked this inhibitory effect.

**Figure 8:**
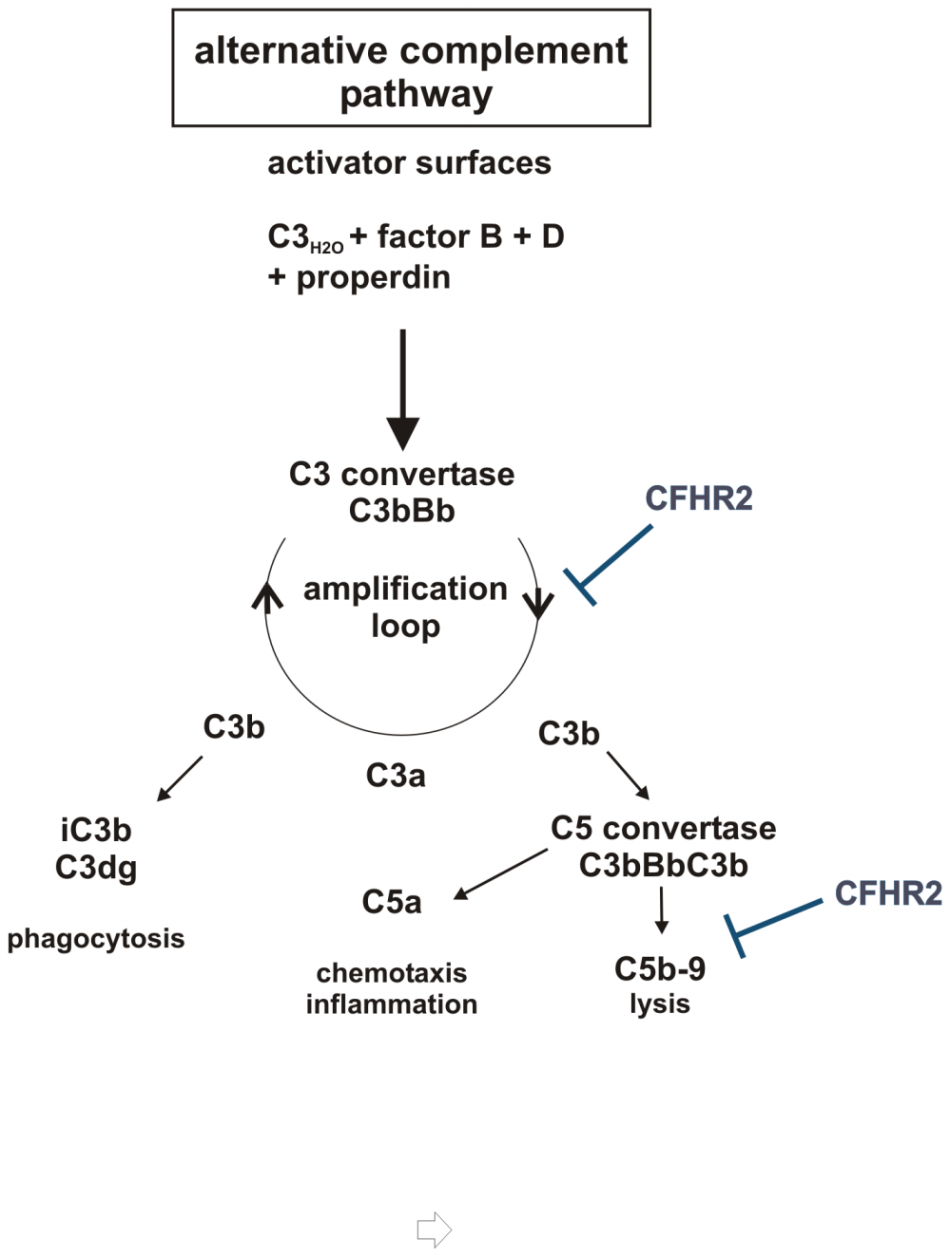
Scematic overview of CFHR2 functions in regulation of the AP. CFHR2 inhibits the amplification loop by inhibiting C3 cleavage by C3 convertases and acts on the assembly of the terminal complement complex.

Compared to factor H, the CFHR2 homodimer binds with about 10 fold lower affinity to C3b and did not efficiently compete for factor H binding to C3b or to C3d. This effect is unique for CFHR2, as both CFHR1- and CFHR5 homo-, as well as CFHR1-CFHR5 heterodimers have been reported to act as competitors for factor H [9]. Thus the C3 convertases to which the CFHR2 homodimer is bound is apparently still accessible for the regulator factor H, which can bind via SCRs 1–4, dissociates Bb from the convertases and displays cofactor activity for factor I. CFHR2 lacks cofactor activity for factor I mediated cleavage and inhibition of C3b. In addition also C3b to which the CFHR2 homodimer is bound can still be processed and inactivated by factor H and factor I to iC3b. Binding of the two differently acting complement regulators CFHR2 and factor H to C3 convertases may ensure a fast and efficient control and inactivation of complement. This is in agreement with the enhanced complement inhibitory effect observed when both regulators together were added to the NHS. In summary CFHR2 is a novel complement regulator that binds to C3b and to the C3 convertase, that controls the C3 convertase of complement and that also inhibits the terminal complement pathway (Fig. 8).
